# Quorum Sensing Controls Both Rhamnolipid and Polyhydroxyalkanoate Production in *Burkholderia thailandensis* Through ScmR Regulation

**DOI:** 10.3389/fbioe.2020.01033

**Published:** 2020-09-04

**Authors:** Sarah Martinez, Adeline Humery, Marie-Christine Groleau, Eric Déziel

**Affiliations:** Centre Armand-Frappier Santé Biotechnologie, Institut National de la Recherche Scientifique (INRS), Laval, QC, Canada

**Keywords:** biosurfactants, *Burkholderia thailandensis*, PHA granules, quorum sensing, gene regulation, metabolites

## Abstract

Rhamnolipids are surface-active agents of microbial origin used as alternatives to synthetic surfactants. *Burkholderia thailandensis* is a non-pathogenic rhamnolipid-producing bacterium that could represent an interesting candidate for use in commercial processes. However, current bioprocesses for rhamnolipid production by this bacterium are not efficient enough, mainly due to low yields. Since regulation of rhamnolipid biosynthesis in *B. thailandensis* remains poorly understood, identifying new regulatory factors could help increase the production of these valuable metabolites. We performed a random transposon mutagenesis screening to identify genes directing rhamnolipid production in *B. thailandensis* E264. The most efficient rhamnolipid producer we identified harbored an inactivating transposon insertion in the *scmR* gene, which was recently described to encode as a secondary metabolite regulator in *B. thailandensis*. We investigated the impact of *scmR* loss on rhamnolipid biosynthesis and cell growth. Because biosynthesis of rhamnolipids and polyhydroxyalkanoates (PHAs) could share the same pool of lipid precursors, we also investigate the effect of ScmR on PHA production. We found that production of both rhamnolipids and PHAs are modulated by ScmR during the logarithmic growth phase and demonstrate that ScmR downregulates the production of rhamnolipids by affecting the expression of both *rhl* biosynthetic operons. Furthermore, our results indicate that PHA biosynthesis is reduced in the *scmR*- mutant, as ScmR promotes the transcription of the *phaC* and *phaZ* genes. By studying the relationship between ScmR and quorum sensing (QS) regulation we reveal that QS acts as an activator of *scmR* transcription. Finally, we pinpoint the QS-3 system as being involved in the regulation of rhamnolipid and PHA biosynthesis. We conclude that ScmR negatively affects rhamnolipid production, whereas it positively impacts PHAs biosynthesis. This could provide an interesting approach for future strain engineering, leading to improved yields of these valuable metabolites.

## Introduction

Rhamnolipids are biosurfactants that were first identified in *Pseudomonas aeruginosa* cultures (Jarvis and Johnson, 1949). The amphiphilic character of rhamnolipids is due to the presence of a hydrophilic polar head, typically containing one or two rhamnose moieties, and a hydrophobic apolar tail, composed of a dimer of two esterified β-hydroxy-fatty acids. This diverse group of compounds comprises more than 60 reported congeners (Abdel-Mawgoud et al., 2010). Variations in the sugar and in the hydrophobic tail lead to structural differences between congeners. Their tensioactive properties, low toxicity and high biodegradability make them suitable for a variety of applications with a low environmental impact (Lang and Wullbrandt, 1999; Banat et al., 2000; Nitschke et al., 2005).

Rhamnolipid biosynthesis has been extensively investigated and the opportunistic pathogen *P. aeruginosa* remains the principal producing bacterial species. However, we previously showed that *Burkholderia thailandensis* also naturally synthesizes rhamnolipids and might represent an interesting candidate for industrial scale production of this biosurfactant (Dubeau et al., 2009). Indeed, this bacterium is non-pathogenic and produces one main congener, facilitating downstream processing. *B. thailandensis* was also recently found to be a producer of polyhydroxyalkanoates (PHAs), another valuable metabolite (Funston et al., 2017; Kourmentza et al., 2018; Martinez and Déziel, 2020) and PHA biosynthesis was suggested to be in metabolic competition with rhamnolipid production (Funston et al., 2017). Production processes for both rhamnolipids and PHAs have not yet been optimized in *B. thailandensis* likely because little is known on the regulation. Two *rhl* operons are responsible for rhamnolipid biosynthesis in *B. thailandensis* (Dubeau et al., 2009). While the global level of transcription of *rhl* genes was studied for the temperature effect on the rhamnolipid production (Funston et al., 2016), the respective regulation of these two operons has yet to be thoroughly explored.

Quorum sensing (QS) is a global regulatory mechanism of gene expression depending on bacterial density (Fuqua et al., 1994). Briefly, LuxI type synthases are responsible for the production of *N*-acyl-L-homoserine lactones (AHLs), signaling molecules which accumulate in the environment during bacterial growth until a threshold concentration. Then, the LuxR type transcriptional regulators are activated by the binding of cognate AHLs, allowing the regulation of the expression of QS target genes including the gene encoding the AHL synthase, creating an autoinducing loop. This way, bacteria synchronize their activities and act as multicellular communities in order to perform social functions. In *B. thailandensis*, the QS systems are referred to as the BtaI1/BtaR1 (QS-1), BtaI2/BtaR2 (QS-2), and BtaI3/BtaR3 (QS-3) systems (Majerczyk et al., 2013), mainly associated with C_8_-HSL, 3OHC_10_-HSL, and 3OHC_8_-HSL, respectively (Chandler et al., 2009; Le Guillouzer et al., 2017). These systems are intricately intertwined and differently regulated depending on the growth phase (Le Guillouzer et al., 2017) and regulate cell auto-aggregation and production of secondary metabolites such as antibiotics (Chandler et al., 2009). In *P. aeruginosa* and in *Burkholderia glumae*, *rhl* biosynthesis genes are under the control of distinct quorum sensing (QS) systems (Soberón-Chávez et al., 2005; Majerczyk et al., 2014a; Nickzad and Déziel, 2016). Although a relationship between QS and the biosynthesis of rhamnolipids has been previously noted in *B. thailandensis* (Majerczyk et al., 2014b; Irorere et al., 2019), there is very limited information on how QS regulates rhamnolipid production.

The aim of this study was to better understand the regulation of rhamnolipid and PHA production in *B. thailandensis* using a mutagenesis and functional screening approach. In doing so, we identified ScmR as a modulator of the biosynthesis of these metabolites in *B. thailandensis*. Since this regulator was also reported to be QS-controlled and to impact the production of AHL signals (Majerczyk et al., 2014a, b; Mao et al., 2017; Le Guillouzer et al., 2020), we further investigated the co-modulation of the biosynthesis of PHA and rhamnolipids by QS and ScmR in *B. thailandensis*.

## Materials and Methods

### Strains and Plasmids

Strains used in this study are presented in Table 1.

**TABLE 1:** Strains and plasmids used in this study.

Strains	Characteristics	References
*Escherichia coli* DH5α	*fhuA2*Δ(*argF-lacZ*)*U169 phoA glnV44*Φ*80*Δ(*lacZ*)*M15 gyrA96 recA1 relA1 endA1 thi-1 hsdR17*	Woodcock et al., 1989
*Burkholderia thailandensis* E264	Wild type strain	Brett et al., 1998
ED1023	E264 *scmR*:pUT-mini-Tn*5*-Km; Km^R^	Le Guillouzer et al., 2020
*B. thailandensis ocb1-* mutant ED3008	E264 BTH_II1071-151:ISlacZ hah, Tc^R^	Gallagher et al., 2013
JBT112	E264 Δ*btaI1*Δ*btaI2*Δ*btaI3*	Chandler et al., 2009
JBT107	E264 Δ*btaR1*	Chandler et al., 2009
JBT108	E264 Δ*btaR2*	Chandler et al., 2009
JBT109	E264 Δ*btaR3*	Chandler et al., 2009
JBT101	E264 Δ*btaI1*	Chandler et al., 2009
JBT102	E264 Δ*btaI2*	Chandler et al., 2009
JBT103	E264 Δ*btaI3*	Chandler et al., 2009
**Plasmids**		
pIT2	Suicide vector IS*lacZ*/hah transposon, Tc^R^	Jacobs et al., 2003
pGP704N-*dfr*	Source of the trimethoprim resistance cassette, Tmp^R^	Lesic and Carniel, 2005
pTZ110	Broad host range *lacZ* fusion vector, Amp^R^	Schweizer and Chuanchuen, 2001
pGEM-T Easy	Cloning vector, Amp^R^	Promega
pAH1	Promoter region of *rhlA1* inserted in pGEM-T Easy	This study
pAH2	Promoter region of *rhlA2* inserted in pGEM-T Easy	This study
pMCG11	pTZ110:*dhfRII*, Tmp^R^	This study
pAH5	Promoter region of *rhlA1* inserted in pMCG11, Tmp^R^	This study
pAH8	Promoter region of *rhlA2* inserted in pMCG11, Tmp^R^	This study
pME6000	Broad-host-range cloning vector; Tc^R^	Maurhofer et al., 1998
pJPD03	*scmR* gene inserted in *Bam*HI-*Hin*dIII sites in pME6000; Tc^R^	Le Guillouzer et al., 2020

*Tc, tetracycline; Tmp, trimethoprim, Bla, beta-lactamase, Km, kanamycin.*

### Growth Conditions

Strains were routinely grown from frozen stocks at 37°C in tryptic soy broth (TSB) (BD Difco) in a TC-7 roller drum at 240 rpm (New Brunswick, Canada), or on TSB agar plates. For rhamnolipid production, nutrient broth (NB) medium (BD Difco) supplemented with 2 or 4% (w/v) glycerol was used (Dubeau et al., 2009). When necessary, antibiotics were used at the following concentrations: 10 μg ml^–1^ tetracycline (Tc) and 100 μg ml^–1^ trimethoprim (Tmp) for *B. thailandensis* and 15 μg ml^–1^ tetracycline (Tc), 100 μg ml^–1^ carbenicillin (Cb), and 100 μg ml^–1^ trimethoprim (Tmp) for *E. coli*. All experiments were performed in triplicate and conducted at least twice independently. For blue/white screening, 5-bromo-4-chloro-3-indolyl-β-D-galactopyranoside (X-gal) was added in LB plates for final concentration 40 μg ml^–1^. For experiments with AHL complementation, 2 μM C_8_-HSL, 3OHC_10_-HSL, or 3OHC_8_-HSL (Sigma-Aldrich Co., Oakville, ON, Canada) were added to cultures. AHLs stocks were prepared in HPLC-grade acetonitrile. Acetonitrile only was added to the controls.

### Random Mutagenesis

A library of *B. thailandensis* transconjugants was generated as follows. Plasmid pIT2 carrying the IS*lacZ*/hah transposon was transferred in *B. thailandensis* E264 by conjugation with *E. coli* χ7213 (*asd*-) strain (Kang et al., 2002; Jacobs et al., 2003). NB agar supplemented with 4% glycerol and tetracycline was used for selection of transposants. The screening for rhamnolipid production was achieved using atomized mineral oil spraying (Burch et al., 2010), with a few modifications. Sudan Red dye (0.5%) was added to mineral oil to provide a better contrast. The presence of a halo surrounding colonies indicates the production of rhamnolipids caused by the amphiphilic properties of surfactants; the diameter of halos around colonies was measured and compared to a WT control. Clones with larger halos were selected as potential candidates for enhanced rhamnolipid production.

#### Identification of Transposon Insertion Sites by Sequencing (Tn-seq)

Total DNA was extracted from bacterial cultures using a mechanical lysis method, as previously described (Durand et al., 2015). DNA concentrations were estimated using the Quant-iT^TM^ PicoGreen^®^ dsDNA Assay Kit (Invitrogen, Life Technologies, Burlington, ON, Canada) following the instructions of the manufacturer. Total genomic DNA from the selected mutants were pooled together and sent to the McGill University and Génome Québec Innovation Centre for transposon insertion sequencing (MiSeq Illumina). Generated Tn-Seq reads were analyzed as follows: sequences were trimmed in order to remove the 3′ bases from the adaptor used for sequencing; only the 4 last bases from the cassette were conserved: TCAG. All the resulting sequences were alphabetically sorted and clustered. For each cluster, a unique consensus sequence was determined and sequence alignments with *B. thailandensis* E264 genome were performed on www.burkholderia.com, allowing the identification of the insertion site.

### Construction of Plasmids

For pMCG11 construction, the *dhfrII* gene, encoding for a dihydrofolate reductase conferring resistance to trimethoprim, was amplified by PCR from pGP704N-*dfr* using primers dhfrFPstI (5′-AAAACTGCAGATATCTG AGCTGTTGACAATTAATCATCC-3′) and dhfrRPstI (5′-A AAACTGCAGCCACCAAACTTAGTTGATGCGTTCAAGCG-3′) and cloned inside the *Pst*I site in the pTZ110 vector. The construct was transformed in *E. coli* DH5α and trimethoprim was used for selection.

Plasmids containing *lacZ* reporters to evaluate the transcription of both *rhl* operons were constructed. First, for pAH1 and pAH2 constructions, the two promoter regions and the first 102 pb of BTH_II1081 (*rhlA1*) and BTH_II1875 (*rhlA2*) genes were amplified by PCR using forward primers Thai-UpOp1-F (5′-GGAATTCCCCGAAGGATATCGGTTTTT-3′) for *rhlA1* and Thai-UpOp2-F (5′-CCGGAATTCCGCATTC ACCACAATGGA-3′) for *rhlA2* respectively, and the reverse primer Thai-UpOp-R (5′-CGGGATCCGTTCACGA GGATGACCGTCT-3′). The PCR products were cloned in the pGEM^®^-T Easy (Promega) to generate vectors pAH1 and pAH2. DH5α cells were transformed and positive clones were selected on LB plate containing carbenicillin and X-gal. The pAH1 and pAH2 plasmids were digested by BamH1 (NEB) and *Ecl*136II (Thermo Fisher Scientific) and the 1081 bp and 847 bp fragments respectively were ligated in *Stu*I (NEB) and *Bam*HI (NEB)-digested pMCG11 to generate pAH5 et pAH8. The two reporters were independently transferred either into *B. thailandensis* wild type (WT) or in the *scmR* mutant by electroporation (Dennis and Sokol, 1995).

### β-Galactosidase Activity Assays

β-galactosidase assays were performed as described (Miller, 1972) with some modifications. Normalization of the activity was calculated using colony-forming units (CFU)/mL instead of OD_600_.

### Rhamnolipid Quantification by Liquid Chromatography/Mass Spectrometry (LC/MS)

Rhamnolipid concentrations in cultures were determined by liquid chromatography coupled to tandem mass spectrometry, as we previously described (Dubeau et al., 2009), with some modifications. After six days of growth, 1 mL culture samples were retrieved and the OD_600_ was measured (Nanodrop ND-1000, Thermo Fisher Scientific). The samples were centrifuged at 16,800 × *g* for 10 min to remove the bacteria. A 500 μl sample of supernatant was transferred to an HPLC vial and 500 μl methanol containing 10 mg/L 5,6,7,8-tetradeutero-4-hydroxy-2-heptylquinoline (HHQ-d4) as the internal standard were added. The samples were then analyzed by high-performance liquid chromatography (HPLC; Waters 2795, Mississauga, ON, Canada) equipped with a C8 reverse-phase column (EVO, Phenomenex) using a water/acetonitrile gradient with a constant 2 mmol/L concentration of ammonium acetate (Dubeau et al., 2009). The detector was a tandem quadrupole mass spectrometer (Quattro Premier XE, Waters). Analyses were carried out in the negative electrospray ionization (ESI-) mode.

### Quantification of Polyhydroxyalkanoates

Polyhydroxyalkanoates concentrations were estimated as described previously (Martinez and Déziel, 2020). Briefly, culture samples were collected and centrifuged during 3 min at 16,800 × *g*. Supernatants were discarded, and pellets were suspended in water. Samples were heated at 100°C for 10 min and immediately transferred on ice for 5 min. The samples were then centrifuged for 3 min at 16,800 × *g* and the pellets were suspended in water and transferred to a 96-well microtiter plate. An equal volume of a 0.02% (w/v) Nile Blue (Sigma Aldrich) solution was added to each well. After 4 min of incubation, the intensity of fluorescence was determined using a Cytation 3 multimode plate reader (Biotek), using excitation and emission wavelengths of 490 and 590 nm, respectively.

### Transmission Electron Microscopy (TEM) for Visualization of PHA Granules

Sample preparation and staining were performed as previously described (Martinez and Déziel, 2020). Ultrathin sections (70−100 nm thick) were examined with a Hitachi H-7100 electron microscope with an accelerating voltage of 75 kV.

### Quantitative Reverse-Transcription Polymerase Chain Reaction (qRT-PCR)

Extraction of total RNA from cultures of *B. thailandensis* E264 and *btai123*, *btaI1*, *btaI2*, *btaI3* mutants at 24, 72, and 96 h, cDNA synthesis and amplification were done as before (Le Guillouzer et al., 2020). The *scmR* gene was amplified using primers SLG_qRThmqR_F (5′-CTTCGTATGTGTTGCCGAAC-3′) and SLG_qRThmqR_R (5′-ATGAGACGCGTGTTCAGATG-3′). To evaluate the expression of genes implicated in PHA accumulation, total RNA was extracted from cultures of *B. thailandensis* E264, *scmR* mutants at 24 and 48 h and cDNA synthesis and amplification were performed as previously described. The *phaC* and *phaZ* genes, which respectively encode for the PHA synthase PhaC and the PHA depolymerase PhaZ were amplified with primers phaC_thai_fwd (5′-GATCTGCTGTACTGGAACG-3′), phaC_thai_rev (5′-AGCTTGTTCTCGAGATAGGT-3′), phaZ_BTH_I0973_fwd (5′-TCTCACTGGGACTTCTATCA-3′), and phaZ_BTH_I0973_rev (5′-TGTATTCGTCGTAG AAGCG-3′).

For all experiments, the reference gene was *ndh*, which encodes for an NADH dehydrogenase (Subsin et al., 2007). This gene displayed stable expression in the various genetic contexts (Supplementary Figure S1). The primers used were the same as in our previous studies SLG_qRT_ndh_F (5′-ACCAGGGCGAATTGATCTC-3′) and SLG_qRT_ndh_R (5′-GATGACGAGCGTGTCGTATT-3′) (Le Guillouzer et al., 2017, 2018, 2020). Differences in gene expression between *B. thailandensis* E264 strains were calculated using the 2^−ΔΔCT^ formula (Livak and Schmittgen, 2001).

## Results

### Random Mutagenesis Reveals a Rhamnolipid Overproducer of *B. thailandensis* E264

**FIGURE 1:**
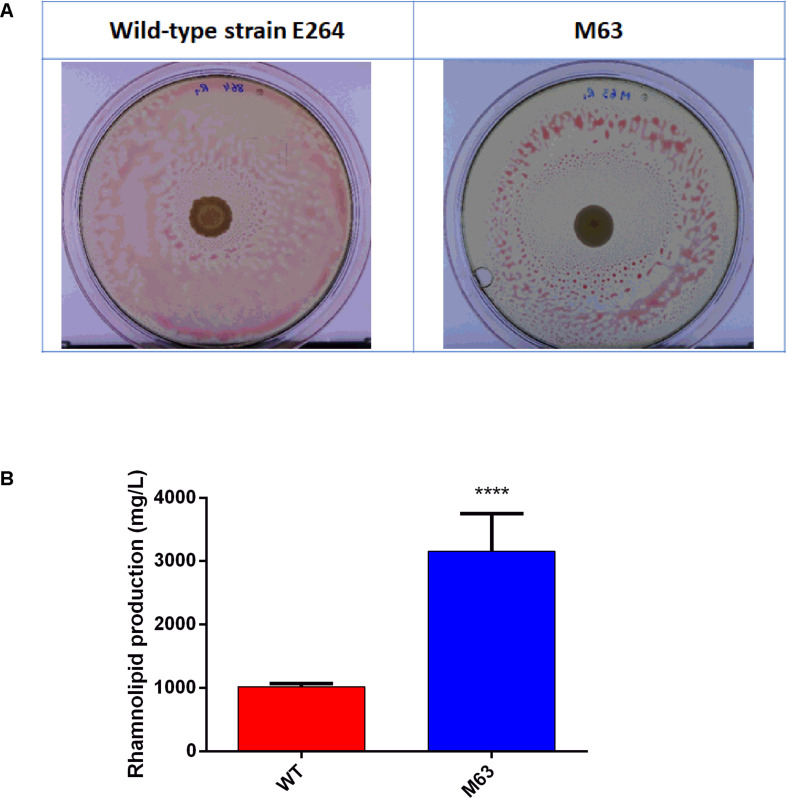
Random mutagenesis identifies a rhamnolipid overproducer of *B. thailandensis* E264. **(A)** Phenotypic selection by oil spraying, **(B)** Rhamnolipid quantification after 5 days of culture in NB + 2% glycerol. The error bars represent standard deviation from the mean (*n* = 3 independent cultures). Asterisk indicates statistically significant differences (*p < 0.05) in paired Student’s *t* tests.

In order to identify factors implicated in the regulation of the production of rhamnolipids in *B. thailandensis*, we performed a random transposon mutagenesis followed by a functional screening. We tested about 25,000 transposants of strain E264 for rhamnolipid production using an oil spraying method. Among the transposants selected, the best candidate for increased rhamnolipid production was isolate M63. It produced a halo of 22.89 ± 3.85 cm^2^, significantly bigger (*t*-test, *p* = 0.0224) than the area around a WT colony, at 6.3 ± 0.21 cm^2^ (Figure 1A). In order to validate the results obtained with the oil spraying method, rhamnolipid production for the M63 mutant was quantified in 5 days-old cultures prepared in NB broth with 2% glycerol. Our results confirmed that the M63 isolate produced three times more rhamnolipids than the WT (Figure 1B).

### A *scmR*- Mutant Overproduces Rhamnolipids

**FIGURE 2:**
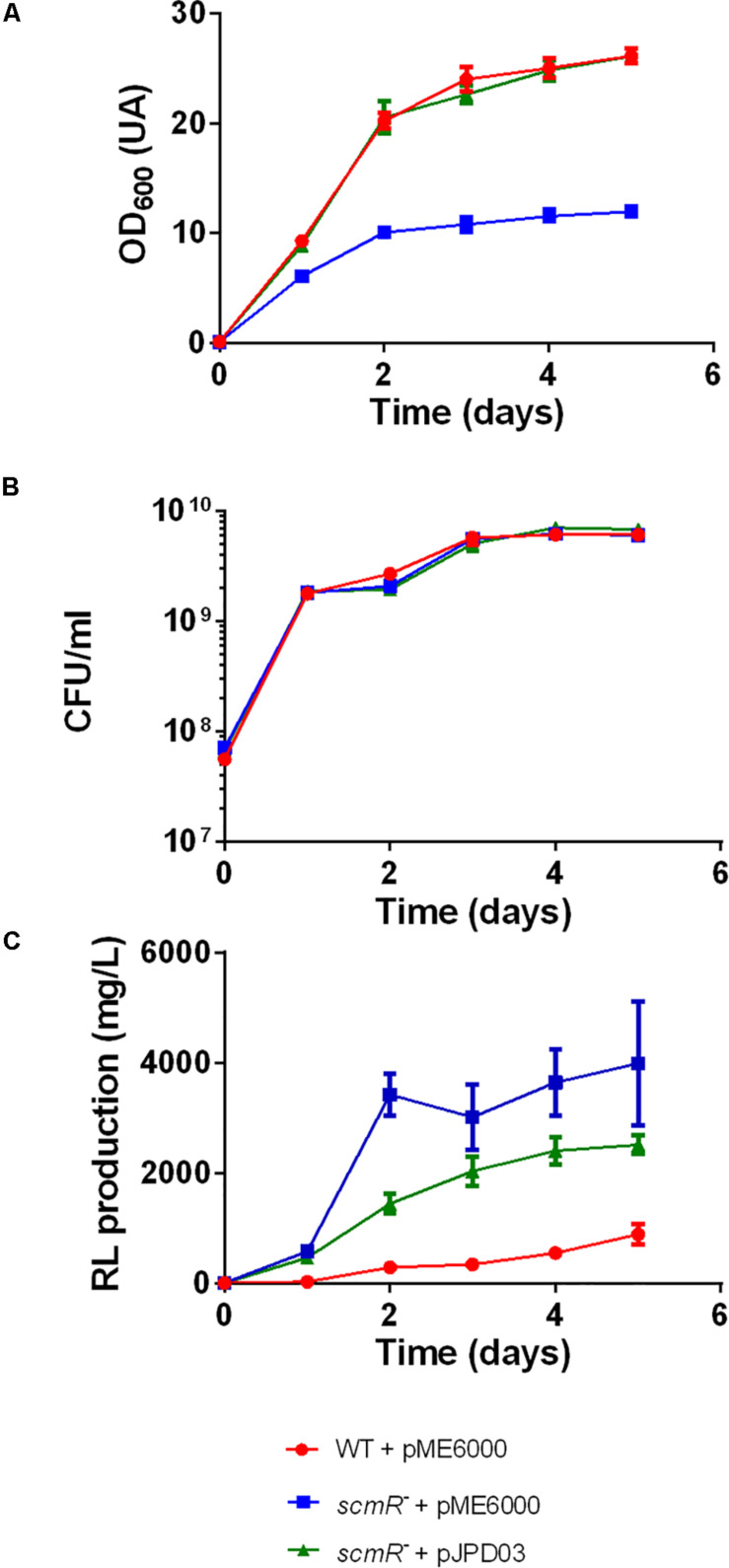
A *scmR* mutant produces more rhamnolipid than the WT strain. **(A)** Growth (OD_600_), **(B)** Growth (CFU/mL), and **(C)** rhamnolipid production (mg/L). The values are means ± standard deviation (error bars) for three replicates.

TnSeq analysis of M63 generated one unique sequence (Supplementary Table S1); this mutant has a transposon inserted in the BTH_I1403 locus, which encodes the LysR-type transcriptional regulator ScmR. Interestingly, this regulator influences the production of several secondary metabolites in *B. thailandensis* E264 (Mao et al., 2017; Le Guillouzer et al., 2020). To investigate the impact of this mutation further, we performed cultures of a previously characterized *scmR-* mutant (ED1023, Table 1; Le Guillouzer et al., 2020) in baffled flasks containing NB medium with 2% glycerol and samples were collected daily. Growth was measured by OD_600_ measurements and live-cell counts (CFU/mL) and rhamnolipid production was quantified by LC-MS analyses. As we observed under different culture conditions (Le Guillouzer et al., 2020), OD_600_ measurements suggested a growth defect for this mutant (Figure 2A). However, CFU counts (Figure 2B) confirmed that both the WT and the *scmR*- mutant actually exhibit identical growth profiles. Rhamnolipid production was confirmed to rapidly reach about 10 times the WT levels within 2 days to finally reach a plateau (Figure 2C). A similar pattern was observed for isolate M63 (Supplementary Figure S2). Complementation of the *scmR* mutant with a plasmid-borne *scmR* gene restored the production toward WT levels (Figure 2C). Partial complementation is not unusual and can be attributed to several factors. First, an indirect regulation of rhamnolipid production is highly possible, thus complementation would also depend on additional regulatory elements. Second, a constitutive, not the native promoter is driving *scmR* on the expression plasmid, precluding transcription matching the appropriate level and timing. Third, ScmR is a LysR-type transcriptional regulator, thus requiring a ligand, whose availability might not be optimal, or depend on ScmR itself, since we reported that it is autoregulated (Le Guillouzer et al., 2020).

### Transcriptions of Both *rhl* Operons Are Augmented in a *scmR-* Mutant

**FIGURE 3:**
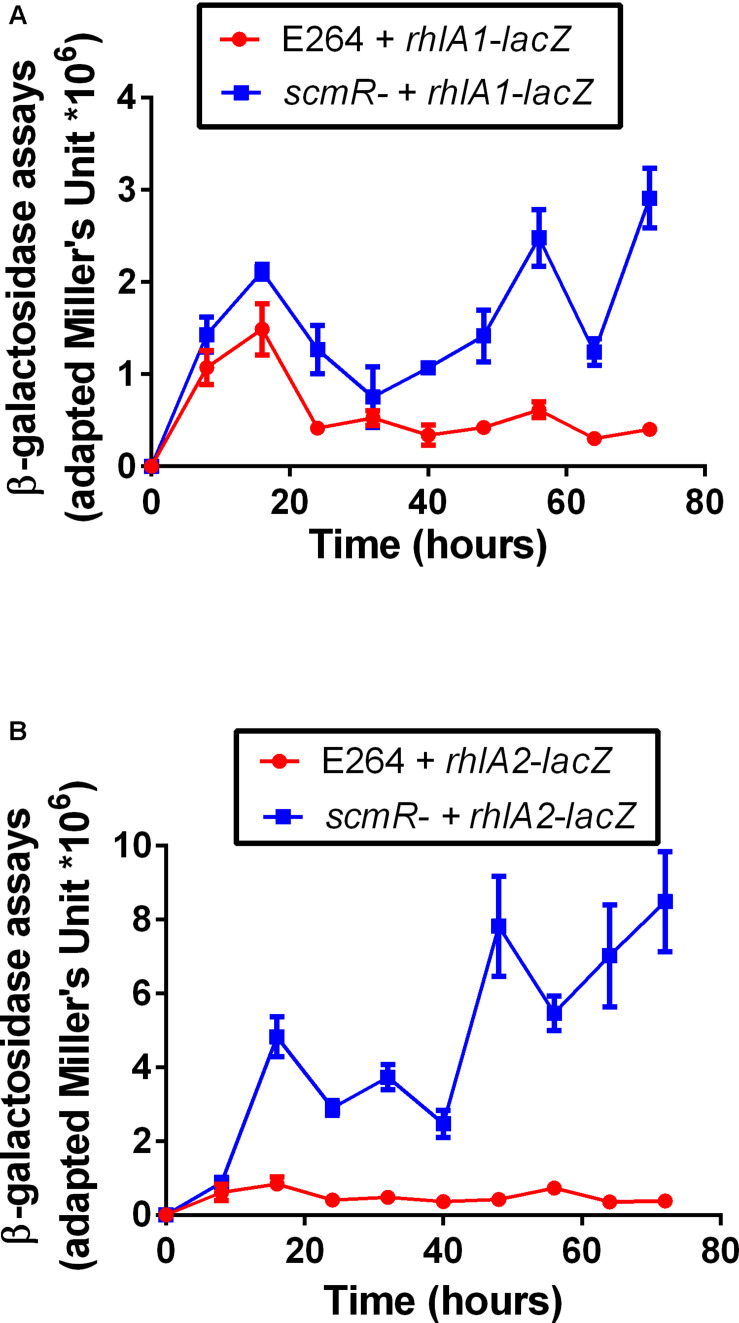
Transcription of both *rhl* operons is increased in the *scmR* mutant. β-galactosidase activity was measured using **(A)** *rhlA1-lacZ* and **(B)** *rhlA2-lacZ* reporters in both the wildtype E264 and the isogenic *scmR* mutant ED1023 in NB medium supplemented with 2% glycerol. OD_420_ measurements were normalized by CFU/mL. The values are means ± standard deviation (error bars) for three replicates.

In *B. thailandensis*, two paralogous *rhl* operons are encoded and functional, both contributing to total rhamnolipid production (Dubeau et al., 2009). We thus verified the expression of each *rhl* operon in both the WT and the *scmR*- mutant ED1023 using transcriptional reporters. While the coding regions of the *rhl* operons are essentially identical, their respective promoter regions are not. Cultures of both the WT and the *scmR*- mutant ED1023 containing a *rhlA1-lacZ* or a *rhlA2-lacZ* reporter were performed in NB medium with 2% (v/v) glycerol. Figure 3 shows that the expression of each operon is higher in the *scmR-* background compared to the WT, revealing that ScmR acts a repressor of both *rhl* operons in strain E264. Interestingly, this effect was even more pronounced for the *rhl2* operon, for which the transcription was up to 10 times higher than the in WT, while the transcription level was up to 4 times higher for *rhl1*, after 72 h of cultivation.

### ScmR Positively Activates PHA Biosynthesis

*Burkholderia thailandensis* is a known PHA producer (Funston et al., 2017; Kourmentza et al., 2018). Indeed, we demonstrated that *B. thailandensis* synthesizes significant levels of PHAs, leading to presence of granules inside the cells which makes unreliable the use of OD_600_ to estimate growth of this bacterium under conditions promoting PHA production (Martinez and Déziel, 2020). Judging by the discrepancy observed between OD_600_ measurements and the CFU counts profiles for both the WT and *scmR*- mutant strains (Figures 2A,B), and since PHAs are secondary metabolites, we hypothesized that ScmR could also affect the production of this biopolymer.

**FIGURE 4:**
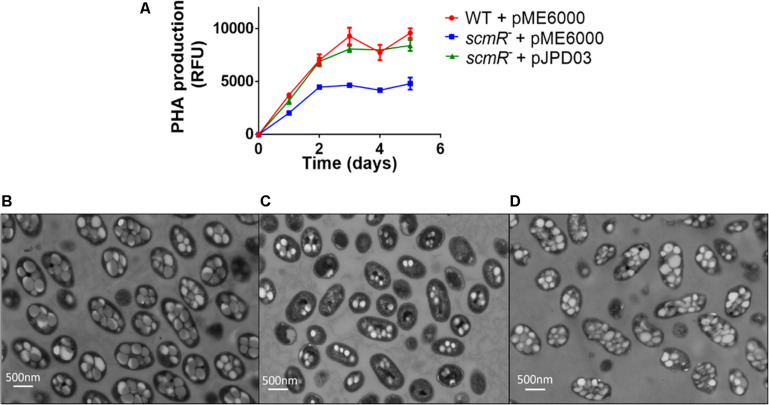
ScmR positively affects PHA biosynthesis. **(A)** PHA levels were determined by fluorescence following Nile Blue staining. The values are means ± standard deviation (error bars) for three replicates. **(B–D)** represent TEM images of E264, the *scmR-* mutant and the complemented *scmR*- mutant, respectively at a magnification of 10,000X.

In order to verify if differences in PHA production are responsible for affecting the OD_600_ measurements shown in Figure 2A, we measured PHA cellular levels with Nile Blue fluorescent staining. PHA production was indeed lower (by about 50%) in the *scmR*- mutant vs the WT (Figure 4A). We then confirmed these results using electron microscopy and found that E264 contained an average of nine granules of PHA per cell, whereas the *scmR-* mutant had much fewer (an average of four granules) (Figures 4B,C). Also, cell shape and length comparisons highlighted that WT cells were 1.4-fold larger than mutant cells. These results revealed that ScmR promotes the production of PHAs in *B. thailandensis* and that PHA accumulation affects the cellular physiology, explaining the apparent growth defect of the *scmR-* mutant (Figure 2A). Complementation of the *scmR*-negative mutant with a plasmid-borne *scmR* gene corrected the OD_600_ difference we observed (Supplementary Figure S3) and restored production of PHAs to WT levels (Figures 4A,D).

### Transcription of the Genes Responsible for the PHA Biosynthesis Is Affected in a *scmR*- Mutant

**FIGURE 5:**
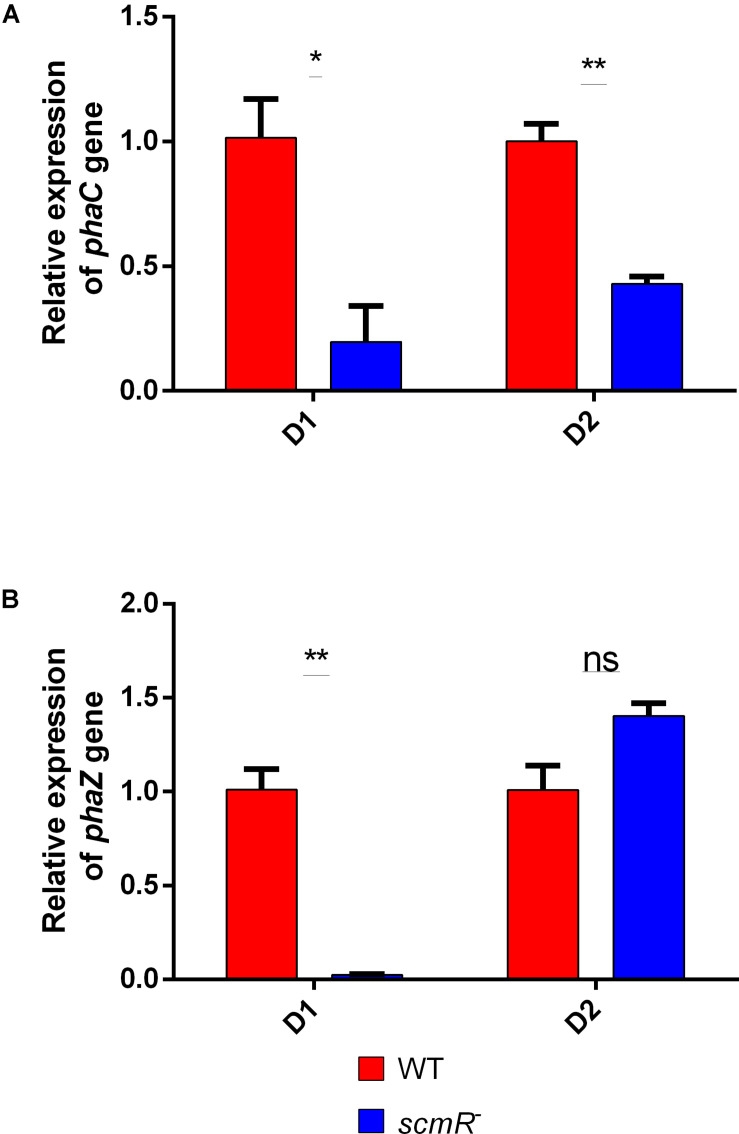
ScmR plays a role in PHA biosynthesis at the transcriptional level. Levels of transcription for the *phaC* **(A)** and *phaZ* **(B)** genes were determined by RT-qPCR in the wild type and the *scmR*^–^ mutant strains. D1 and D2 are days of cultivation. The error bars represent standard deviation from the mean (*n* = 3 independent cultures). Data analyzed using a one-way ANOVA with *post hoc.* Dunnett’s multiple comparisons tests (^∗∗^*p* < 0.01, ^∗^*p* < 0.05, and ns = not significant).

In order to better understand the effect of ScmR on the production of PHA, we looked at the expression of two genes implicated in PHA production. We previously demonstrated that the *phaC* gene from *B. thailandensis*, which encodes the PHA synthase, is homologous to the one found in the *Cupriavidus* genus (Martinez and Déziel, 2020). Also, in *Cupriavidus necator*, other genes are playing a role in PHA production, such as *phaZ*. The transcription levels of *phaC* and *phaZ* were quantified by qRT-PCR in the *B. thailandensis* E264 and in the *scmR-* mutant grown in NB with 2% glycerol, at 24 and 48 h of incubation. We measured reduced expression of *phaC* at both time points (Figure 5A), which correlates with the production patterns we observed (Figure 4A). The transcription levels of *phaZ* were also lower in the *scmR* mutant after 24 h, compared to the WT strain, while no significant difference was seen at 48 h (Figure 5B). All together, these data indicate that the decrease in PHA production we observed in the *scmR* mutant was likely caused by a lowered expression of *pha* metabolic genes.

### The Expression of ScmR Is Regulated by Quorum Sensing

We demonstrated that ScmR is involved in both rhamnolipid and PHA biosynthesis. QS activates the transcription of the *scmR* gene in cultures of *B. thailandensis* grown in TSB (Le Guillouzer et al., 2020). Also, concentrations of all three AHLs are reduced in a *scmR*- mutant compared to the WT (Mao et al., 2017) when *B. thailandensis* is cultured in LB medium. We thus investigated the interplay between ScmR and QS to verify if they are associated in regulating rhamnolipid and PHA production under our specific conditions.

**FIGURE 6:**
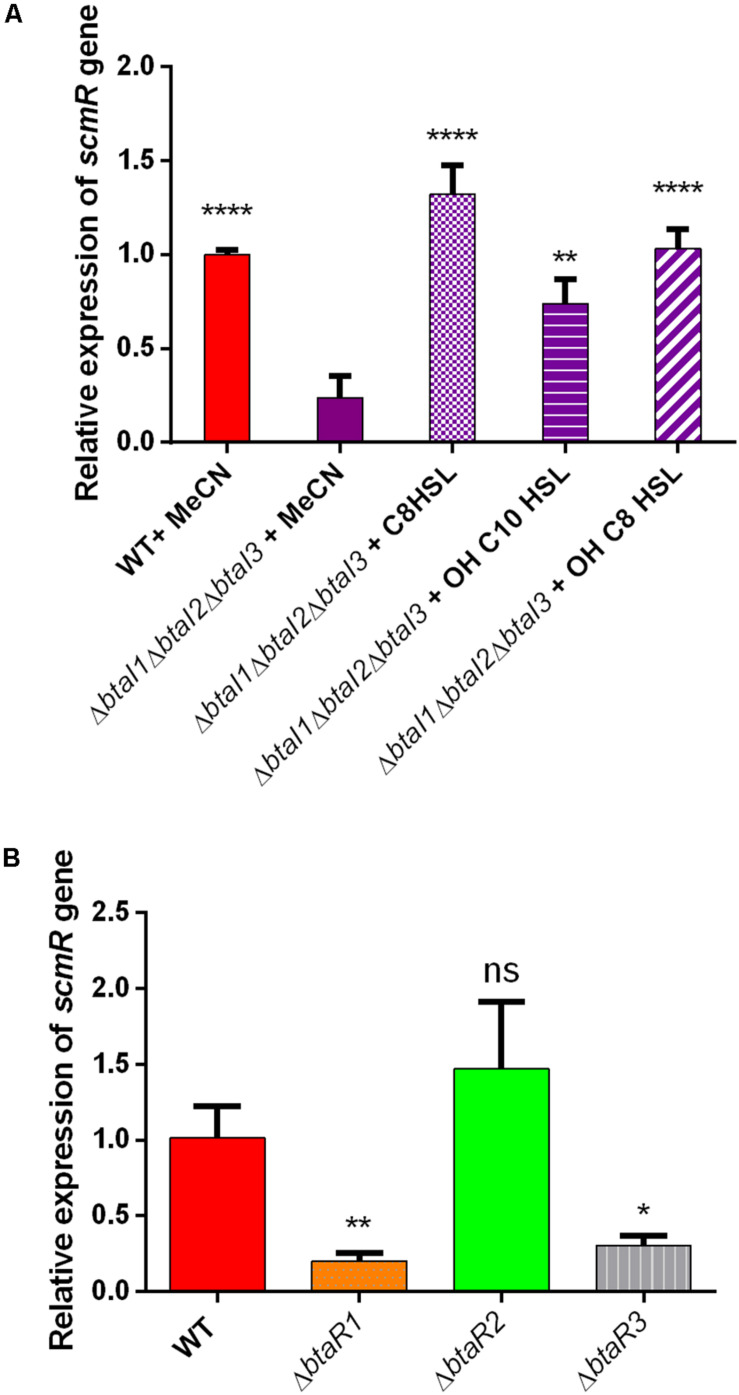
QS-1 and QS-3 activate the transcription of *scmR*. **(A)** The relative transcript levels of *scmR* in the *B. thailandensis* E264 wild-type and the Δ*btaI1*Δ*btaI2*Δ*btaI3* mutant strains were determined by qRT-PCR. Cultures were supplemented with 2 μM of C_8_-HSL, 3OHC_10_-HSL, or 3OHC_8_-HSL. Acetonitrile (MeCN) only was added to the controls. The results are presented as relative quantification of transcription of the gene compared to the wild-type strain, which was set at 100%. **(B)** The relative transcript levels of *scmR* were assessed by qRT-PCR in cultures of the wild-type and of the Δ*btaR1*,Δ*btaR2*, and Δ*btaR3* mutants of strain E264. The error bars represent standard deviation from the mean (*n* = 3 independent cultures), Data analyzed using a one-way ANOVA followed by Dunnett’s multiple comparisons tests (^****^p < 0.0001, ^∗∗^*p* < 0.01, ^∗^*p* < 0.05, and ns = not significant).

To further investigate the regulation of *scmR* by QS in NB with 2% glycerol, the transcription levels of *scmR* were quantified by qRT-PCR in the *B. thailandensis* E264 WT strain vs the Δ*btaI1*Δ*btaI2*Δ*btaI3* mutant. Since *B. thailandensis* possesses three QS systems (QS-1, QS-2 and QS-3) mainly associated with the production of C_8_-HSL, 3OHC_8_-HSL and 3OHC_10_-HSL respectively, we supplemented (or not) the AHL-defective Δ*btaI1*Δ*btaI2*Δ*btaI3* mutant with exogenous AHLs during the logarithmic growth phase to distinguish which of the three QS systems modulates the transcription of *scmR* under these culture conditions. We observed that expression of *scmR* is reduced in the absence of AHLs (Figure 6A), confirming that *scmR* transcription is positively modulated by QS. Furthermore, the transcription of *scmR* was increased in cultures of the Δ*btaI1*Δ*btaI2*Δ*btaI3* mutant when any of C_8_-HSL, 3OHC_10_-HSL, or 3OHC_8_-HSL were provided (Figure 6A). The response was higher with addition of C_8_-HSL and 3OHC_8_-HSL, associated with the QS-1 and QS-3 systems, respectively. The transcription of *scmR* in the Δ*btaR1*, Δ*btaR2*, and Δ*btaR3* mutants and the *B. thailandensis* E264 WT strain was also studied during the logarithmic growth phase in NB with 2% glycerol. No difference in *scmR* transcription was observed in Δ*btaR2*. In contrast, it was decreased in both the Δ*btaR1* and Δ*btaR3* mutants (Figure 6B). Together, these results indicate that both QS-1 and QS-3 upregulate the transcription of *scmR*, but not QS-2.

### Quorum Sensing Modulates the Production of Both Rhamnolipids and PHAs

**FIGURE 7:**
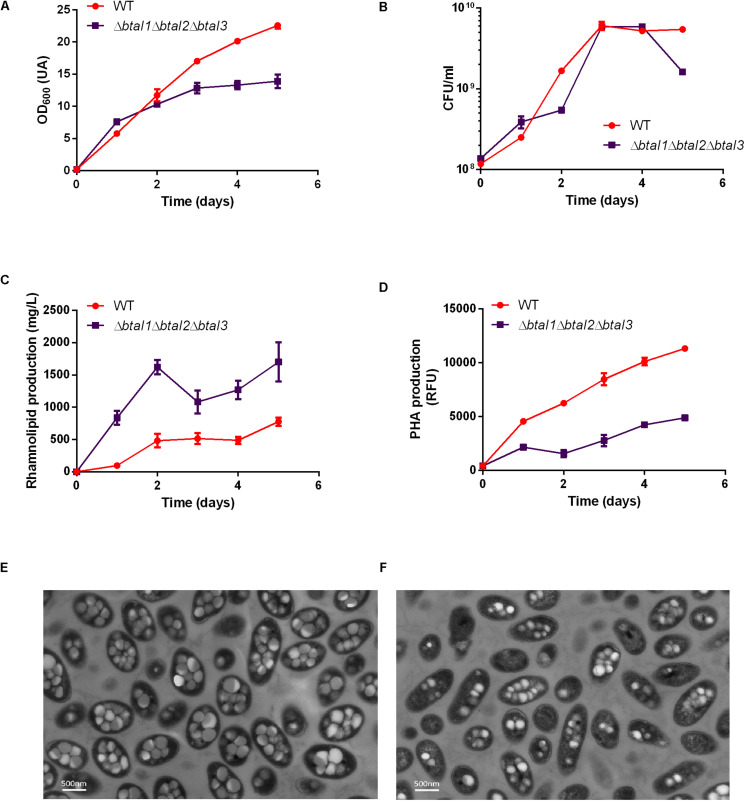
Rhamnolipid and PHA production are quorum sensing-controlled. The wild type strain and the Δ*btaI1*Δ*btaI2*Δ*btaI3* quorum sensing mutant were cultured for five days at 30°C in NB medium supplemented with 2% glycerol. The **(A)** OD_600_, **(B)** CFU/mL, **(C)** PHA levels, and **(D)** rhamnolipid concentration measurements are represented. Values are means ± standard deviations (error bars) for three replicates. TEM images were taken at day 4 for panels **(E)** the wild type strain and **(F)** the Δ*btaI1*Δ*btaI2*Δ*btaI3* mutant.

We then investigated the production of rhamnolipids in the AHL-defective mutant Δ*btaI1*Δ*btaI2*Δ*btaI3* compared to the WT. Because production of rhamnolipids and PHAs are closely related, we also measured PHA levels in the same cultures. Rhamnolipid production was about two times higher in the Δ*btaI1*Δ*btaI2*Δ*btaI3* mutant vs the WT (Figure 7C). On the other hand, the Δ*btaI1*Δ*btaI2*Δ*btaI3* mutant produced less than half the PHAs compared to the WT (Figure 7D) and this was confirmed by TEM images of cells sampled at day 4 (Figures 7E,F). Accordingly, we observed a difference in OD_600_ measurements for the Δ*btaI1*Δ*btaI2*Δ*btaI3* mutant when compared to WT (Figure 7A), but once again CFU counts confirmed that growth was similar between both strains (Figure 7B). The exact same situation was observed with the *scmR*- mutant. We thus rationalized that the QS-null strain mostly behaves like the *scmR*- mutant because QS activates *scmR* (Figure 7A).

**FIGURE 8:**
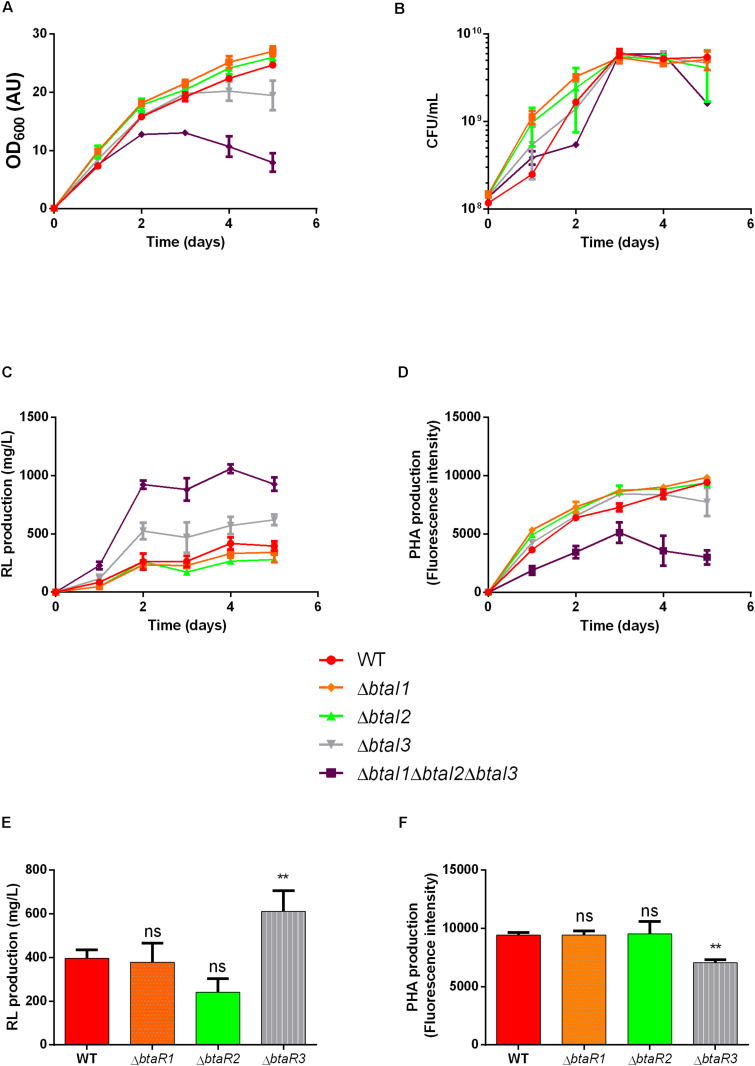
The QS-3 quorum sensing system affects the production of both PHAs and rhamnolipids. Wild type strain E264 and isogenic quorum sensing mutants were cultured during five days at 30°C in NB medium supplemented with 2% glycerol. Growth measurements are described for Δ*btaI* mutants in panel **(A)** OD_600_ and **(B)** CFU/mL. **(C)** Rhamnolipid concentrations and **(D)** PHA levels are presented for the Δ*btaI* mutants. **(E)** Rhamnolipid concentrations and **(F)** PHA levels are presented for the Δ*btaR* mutants in the QS-1, QS-2 and QS-3 systems. The error bars represent standard deviation from the mean (*n* = 3 independent cultures), Data analyzed using a one-way ANOVA with *post hoc.* Dunnett’s multiple comparisons tests (^∗∗^*p* < 0.01 and ns = not significant).

Finding that loss of *scmR* increases the production of rhamnolipids and decreases the production of PHAs brought us to identify the complex QS circuitry of *B. thailandensis* as an apparently contrasting regulator of production of these metabolites. We then sought to separately investigate the effect of the three QS systems in the regulation of rhamnolipid production in E264. We quantified rhamnolipids and PHAs in cultures of individual *btaI* and *btaR* mutants after 5 days. Interestingly, none of three individual *btaI* mutants replicated the OD_600_ defect of the triple mutant, suggesting no decrease in PHA production, apart from a partial reduction for the *btaI3* mutant (Figure 8A). Accordingly, growth was not affected (Figure 8B). No difference was observed in final rhamnolipid levels when we compared the Δ*btaI1*, Δ*btaI2* or Δ*btaR1*, Δ*btaR2*- mutants to the WT strain E264. In contrast, rhamnolipid concentrations were increased when *btaI3* or *btaR3* were inactivated (Figures 8C,E). Similarly, no significant difference in PHA biosynthesis was observed for QS-1 and QS-2 mutants, while we measured less PHAs in the QS-3 system mutants (Figures 8D,F). Based on these observations, we conclude that the QS-3 system negatively affects rhamnolipid production whereas it positively impacts PHA biosynthesis, similarly to ScmR, but less pronounced.

## Discussion and Conclusion

The non-pathogenic bacterium *B. thailandensis* is a promising candidate for the production of rhamnolipids (Dubeau et al., 2009; Funston et al., 2016; Irorere et al., 2018; Kourmentza et al., 2018). However, current yields remain low. While the biosynthetic genes have been identified, their regulation is poorly understood. We propose that better knowledge of this aspect is important to improve the production of these metabolites. The primary aim of this work was to identify genes involved in the regulation of rhamnolipid biosynthesis. Our functional screening allowed us to identify ScmR as a negative regulatory element of rhamnolipid production. In *B. thailandensis*, global transcription of the *rhl* biosynthetic genes was previously studied (Funston et al., 2016) using qRT-PCR on coding sequences, thus without taking into consideration that two identical *rhl* operons are present in *B. thailandensis* (Dubeau et al., 2009). Both operons distinctly contribute to the total rhamnolipid production. Indeed, a mutation in either *rhlA1* or *rhlA2* causes a decrease but not the abolishment of rhamnolipid biosynthesis. Hence, we previously reported that the *rhlA2* operon is responsible for a larger portion of total rhamnolipid production since the *rhlA1* mutant produces more rhamnolipids than the *rhlA2* mutant (Dubeau et al., 2009). This suggests that both *rhl* operons are differently regulated. Accordingly, the transcription of both *rhl* operons was differently increased in the *scmR-* mutant, at least partially explaining how ScmR represses rhamnolipid biosynthesis. Indeed, the effect on *rhlA2* was larger than on *rhlA1*, showing again that the regulation of both operons is different. Further studies are needed to better understand the distinct regulation of each *rhl* operon. Whether or not the regulation of ScmR on each of the *rhl* operons is direct has yet to be confirmed.

Similarly, PHA production is not well documented in *B. thailandensis*. Only few recent studies reported the production of PHAs in this bacterial species. One of them revealed the potential of PHAs production of strain E264 (Funston et al., 2017). Another one described PHAs produced during growth on used cooking oil-containing medium (Kourmentza et al., 2018), where PHA biosynthesis occurred during the logarithmic phase. In several bacteria, excess carbon availability promotes PHA production when nutritional elements such as N, P or Mg are limiting (Anderson and Dawes, 1990). However, based on our observations, it seems that *B. thailandensis* belongs to the group of growth-associated PHA producers, meaning that limitation of an essential nutrient is not necessary for the induction of PHA biosynthesis. Other *Burkholderia* species can produce PHAs, such as *B. xenovorans*, *B. sacchari*, and *B. cepacia* (Urtuvia et al., 2014), so it is consistent that *B. thailandensis* is also a significant producer. PHA production is regulated by QS in other bacteria such as *Pseudomonas chlororaphis* (Mohanan et al., 2019), *Rhodobacter sphaeroides* (Kho et al., 2003) and *Vibrio harveyi* (Sun et al., 1994). However, QS regulation can be either negative or positive depending on the organism and/or the conditions.

When looking at the transcription of two genes implicated in the biosynthesis of PHAs, we found that the transcription of both *phaC* and *phaZ* was affected in the *scmR-* mutant. When comparing to the sequence of *phaZa*1 from *C. necator* H16, there are two predicted PHA or PHB depolymerase genes in the genome of *B. thailandensis* E264 (BTH_II2312 and BTH_I0973). Sequence comparison analyses show that gene BTH_ I0973 presents the highest identity percentage (79.4%) and we thus consider it as the most likely *phaZ* candidate (Saegusa et al., 2001; Uchino et al., 2008). Our finding that ScmR activates PHA biosynthesis by modulating the transcription of *phaC* and *phaZ* is interesting for future investigations on the potential of *B. thailandensis* as a PHA producer. Whether the positive effect of ScmR on the transcription of *phaC* and *phaZ* is direct or indirect remains to be determined.

The implication of the ScmR regulator in secondary metabolism was recently described in *B. thailandensis*, where it was reported to act as an important repressor (Mao et al., 2017). Our data showed that ScmR regulates the production of rhamnolipids and PHA while neither *rhl* nor *pha* biosynthesis genes were found in transcriptomic studies performed on *scmR*- mutants (Mao et al., 2017; Le Guillouzer et al., 2020). We noted that the LB medium is not appropriate for rhamnolipid production compared to NB medium complemented with 2% glycerol (Dubeau et al., 2009), at least partially explaining the absence of *rhl* gene regulation in the Mao et al. (2017) study. While they characterized the ScmR regulon in the stationary phase (Mao et al., 2017), we confirm here that this regulator is already active during the logarithmic phase. Interestingly, we found that in the WT, both metabolites are synthetized during growth but only the concentration of PHAs keeps augmenting when stationary phase is reached. In contrast, when *B. thailandensis* was cultivated on used cooking oil, rhamnolipid biosynthesis continued after the stationary phase was reached, while PHA production decreased (Kourmentza et al., 2018), suggesting a tight regulation of these two related metabolites.

The slow-down in rhamnolipid production we observed under our conditions at the stationary phase could be because of changes in nutrient availability, in pH, or in dissolved oxygen concentration. For instance, restricted rhamnose availability could explain why rhamnolipid production was reduced while PHAs kept being synthetized using the lipid precursors. In *P. aeruginosa*, dissolved oxygen concentration has a crucial effect on rhamnolipid production (Zhao et al., 2018; Bazsefidpar et al., 2019). The pH was also reported to impact rhamnolipid production in *P. aeruginosa* (Chen et al., 2007; Zhu et al., 2012). In *B. thailandensis* cultures in NB medium with 2% glycerol, the pH increased during the log phase and then decreased at the stationary phase (Supplementary Figure S4A). Oxalate production by Obc1, encoded by the *obc1* gene, is involved in the decrease of pH observed in cultures of *B. thailandensis* (Goo et al., 2012). We know that *obc1* transcription is strongly downregulated in the *scmR-* mutant (Le Guillouzer et al., 2020). Indeed, in cultures of the *scmR-* mutant, pH remained higher than in cultures of the WT and this difference could be responsible for the slowing in rhamnolipid production. To verify this hypothesis, we compared rhamnolipid production in cultures of an *obc1* mutant, unable to produce d-oxalate, vs the WT E264 strain. Rhamnolipid production was not affected in this mutant, suggesting that a pH increase is not involved in the late slow-down in rhamnolipid production (Supplementary Figure S4B).

The QS circuitry of *B. thailandensis* is very complex, including homeostatic regulatory loops and additional regulators (Le Guillouzer et al., 2017, 2018). Production of rhamnolipids and other biosurfactants are often regulated by QS in various bacterial species. An initial global analysis of QS in *B. thailandensis* revealed that it seems to affect the transcription of *rhlA1*, *rhlB1* and *rhlB2*, encoding rhamnolipid biosynthesis (Majerczyk et al., 2014b). However, this study was performed in LB medium, as stated above not ideal to induce *rhl* genes. Plus, no involvement of QS into the regulation of known PHA synthesis genes was described in this transcriptomic study, again suggesting the use of culture conditions not appropriate to induce biosynthesis. The production of both rhamnolipids and PHAs was specifically studied with the Δ*btaI1*Δ*btaI2*Δ*btaI3* QS-defective mutant (Irorere et al., 2019) and was affected. Indeed, rhamnolipid production was increased while PHA biosynthesis was decreased (Irorere et al., 2019) which correlates with our observations. Interestingly, in *B. thailandensis*, QS appears to have an opposite effect on rhamnolipid production compared to in *P. aeruginosa* or *B. glumae*. In *P. aeruginosa*, the disruption of the *rhlR* QS system leads to a deficiency in rhamnolipid production and a functional RhlR is necessary for biosynthesis (Ochsner et al., 1994; Nakata et al., 1998). Similarly, mutation in the TofI/R QS system in *B. glumae* downregulates the production of rhamnolipids (Nickzad et al., 2015).

QS and ScmR are closely linked. Hence, the production of all three AHLs is reduced in a *scmR-* mutant (Mao et al., 2017; Le Guillouzer et al., 2020) which mimics the QS-deficient mutant. The presence of a Lux-box into the *scmR* promoter suggests that QS could directly activate the transcription of ScmR (Mao et al., 2017). Accordingly, we found lower transcription of *scmR* in both Δ*btaR1* and Δ*btaR3* mutants (Figure 5; Le Guillouzer et al., 2020). Moreover, the addition of the three AHLs led to increased *scmR* transcription in a Δ*btaI*1Δ*btaI2*Δ*btaI3*, especially by C_8_-HSL and the 3OHC_8_-HSL which are principally produced by the QS-1 and QS-3 systems, respectively. Interestingly, we previously found an interdependence between the QS-1 and QS-3 systems (Le Guillouzer et al., 2017). Indeed, *btaI3* transcription is controlled by the BtaR1/C_8_-HSL complex during the logarithmic growth phase while BtaR3 could be involved in the modulation of the *btaI1* gene in conjunction with 3OHC_10_-HSL and 3OHC_8_-HSL (Le Guillouzer et al., 2017). Here, we observed a significant augmentation in rhamnolipid production and a decrease in PHA accumulation in the *btaI3*- and *btaR3*- mutants, consistent with *scmR* upregulation by the QS-3 system. In addition, we showed that *scmR* transcription is activated by BtaR1, presumably through an indirect activation of QS-3 by QS-1. These observations concur with our data obtained when *B. thailandensis* is grown in TSB medium (Le Guillouzer et al., 2020). Rhamnolipid production was higher in the *scmR-* mutant compared to the Δ*btaI1*Δ*btaI2*Δ*btaI3-* mutant. Indeed, the Δ*btaI1*Δ*btaI2*Δ*btaI3* strain produced 1.7 times more rhamnolipid compared to the WT strain while the *scmR-* mutant produced about 3-4 times more rhamnolipid than the WT. Similarly, PHA production was reduced by 40% in the Δ*btaI1*Δ*btaI2*Δ*btaI3* and 50% in the *scmR-* mutant. Collectively, these observations suggest that QS is not the only regulation positively affecting the transcription of the *scmR* gene. We conclude that inhibition of rhamnolipids and increase in PHAs production by QS probably go through an effect on ScmR.

**FIGURE 9:**
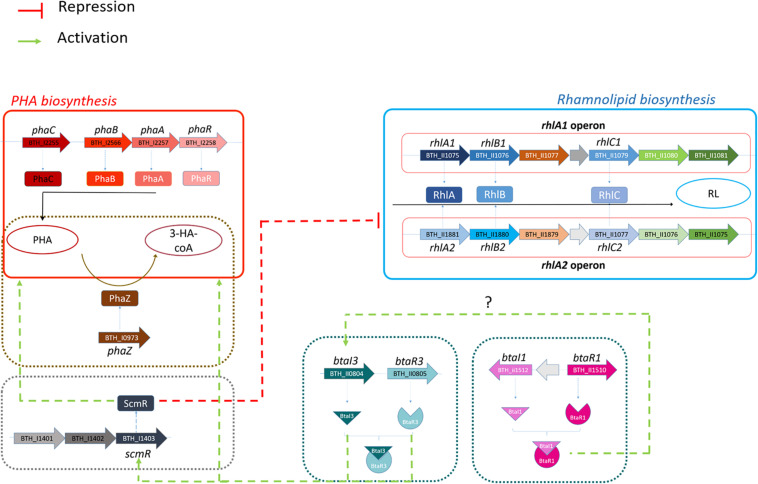
Proposed regulation of rhamnolipid and PHA productions through ScmR and quorum sensing systems in *B. thailandensis* E264. Rhamnolipid and PHA biosynthesis pathways are presented in red and blue, respectively. The involvement of ScmR, QS-1 and QS-3 on rhamnolipid and PHA production are represented using green dotted arrows for activation or red dotted lines for the repression. Dotted arrow lines indicate that direct regulation has not been confirmed.

In conclusion, this study significantly improves our understanding of the regulation of both rhamnolipid and PHA production in *B. thailandensis*. We demonstrate that QS, and most specifically the QS-3 system, directs this production (Figure 9). We also found that ScmR is a negative regulatory element of rhamnolipid production and positively affects PHA synthesis and confirmed the QS-dependent regulation of the *scmR* gene. Since there is a difference in PHA and rhamnolipid production between the Δ*btaI1*Δ*btaI2*Δ*btaI3* and *scmR-* mutants, we conclude that *scmR* is not exclusively modulated by QS, suggesting more work is needed to fully uncover the regulation of rhamnolipid and PHA production in *B. thailandensis* to optimize it to its full potential. ScmR and QS represent promising targets to engineer strains of *B. thailandensis* producing more rhamnolipids or PHAs.

## Data Availability Statement

All datasets generated for this study are included in the article/Supplementary Material.

## Author Contributions

SM, AH, and ED conceived and designed the experiments. SM performed the experiments. SM, AH, M-CG, and ED analyzed the data. AH and M-CG contributed reagents, materials, and analysis tools. SM, M-CG, and ED contributed to writing, editing, and finalizing the manuscript. All authors contributed to the article and approved the submitted version.

## Conflict of Interest

The authors declare that the research was conducted in the absence of any commercial or financial relationships that could be construed as a potential conflict of interest.
