# Supplementary material for: Quorum Sensing Controls Both Rhamnolipid and Polyhydroxyalkanoate Production in Burkholderia thailandensis Through ScmR Regulation
Source: Front Bioeng Biotechnol. 2020 Sep 4;8:1033. doi: 10.3389/fbioe.2020.01033 (PMC7498548; doi:10.3389/fbioe.2020.01033)
Supplement: Supplementary file 1 [file Data_Sheet_1.PDF]

Supplementary material

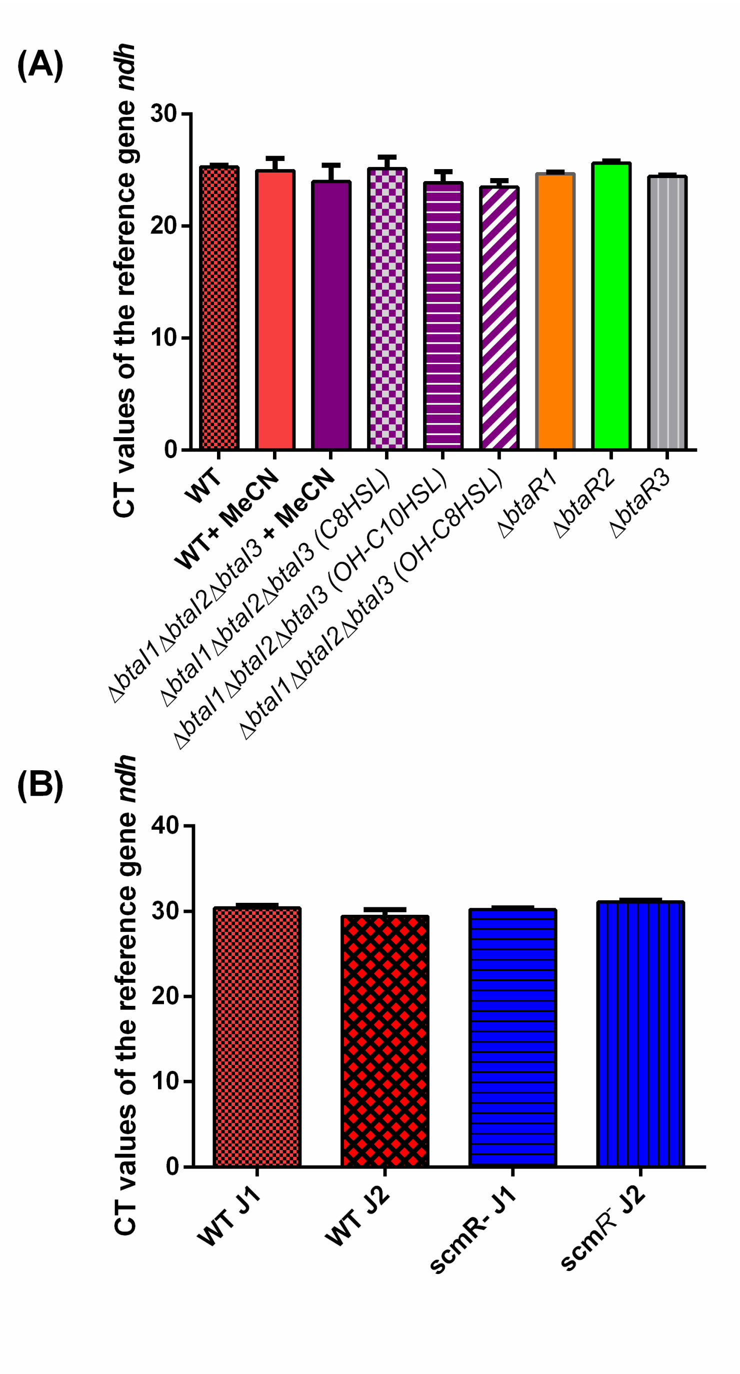

Supplementary figure S1: Treshold cycle (CT) values of the housekeeping gene *ndh.* CT values of the ndh gene used as a reference gene for qRT-PCR experiments are described for the different conditions we tested. (A) Cultures in the wild type *B.thailandensis* E264 and in its QS mutants supplemented with the appropriate AHL when necessary (Acetonitrile was added as a control). (B) Cultures in the wild type *B. thailandensis* E264 and the *scmR*$^-$ mutant strains after one day and two days of cultivation.

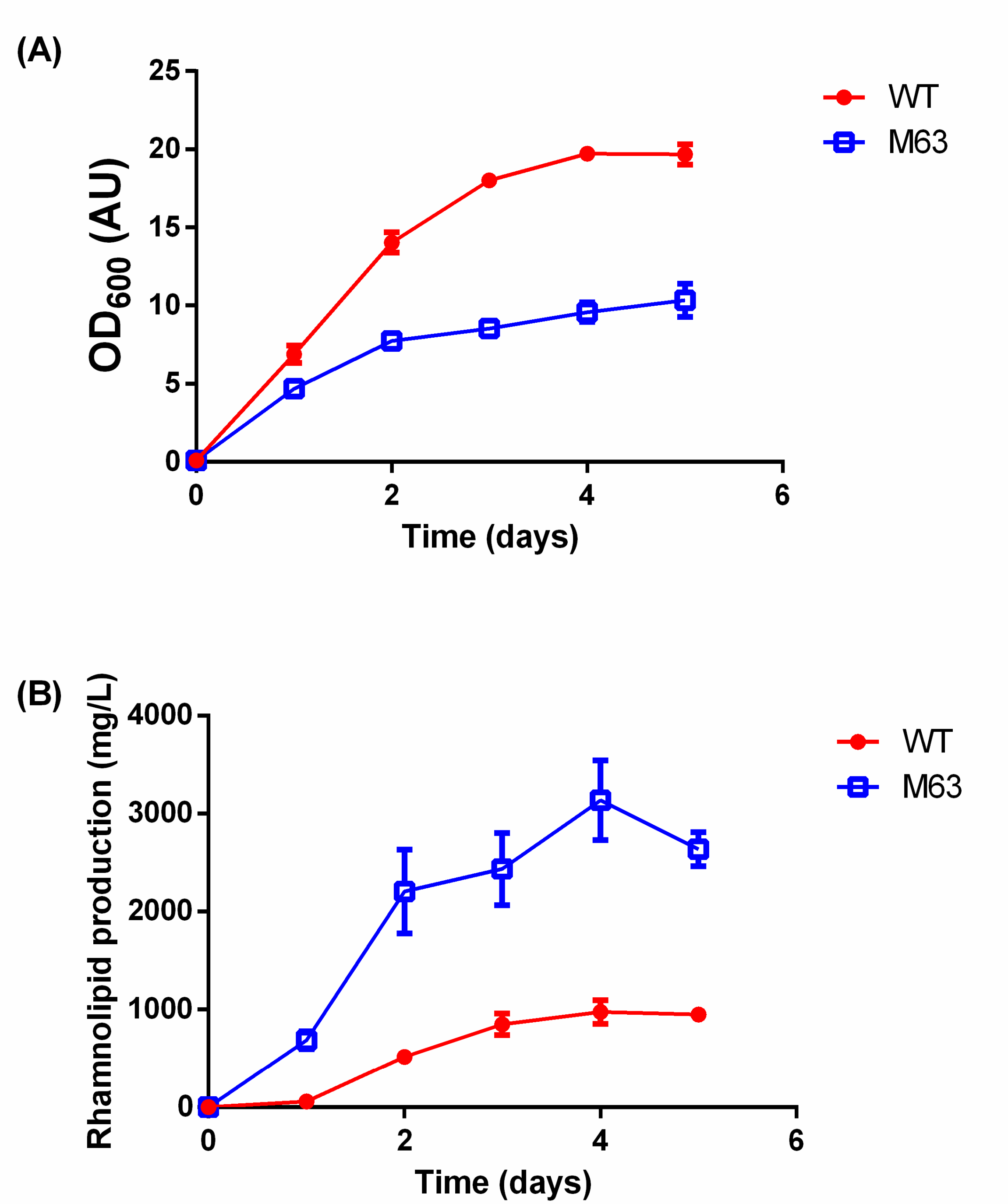

Supplementary Figure S2: The M63 isolate produces more rhamnolipid than the WT strain. (A) Growth (OD_{600}), and (B) rhamnolipid production (mg/L). The values are means ± standard deviations (error bars) for three replicates.

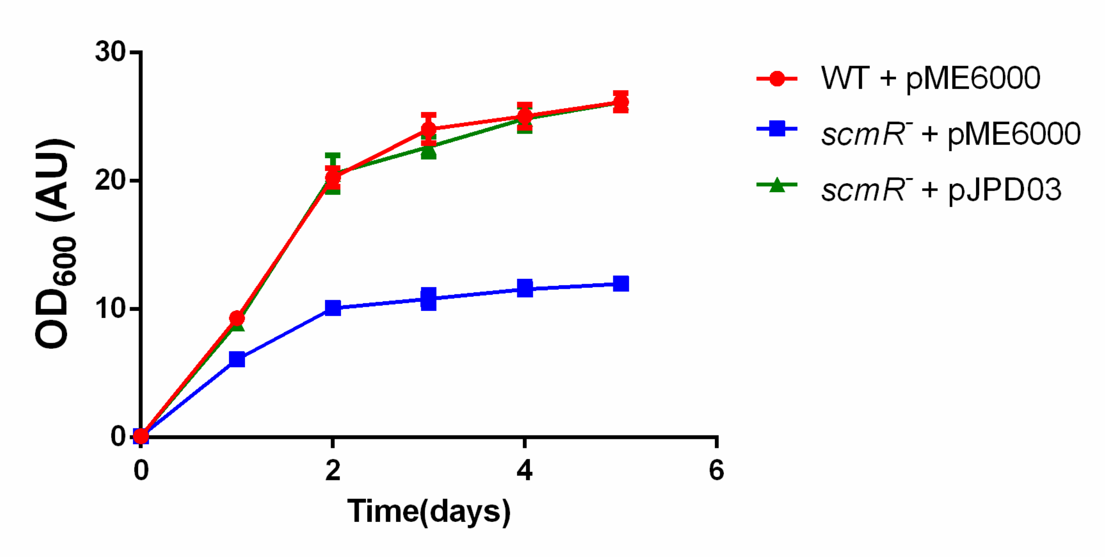

Supplementary Figure S3: Optical density is affected in a *scmR*⁻ mutant. Optical density was measured during 5 days in E264, the *scmR*- mutant and the complemented *scmR*- mutant .The values are means ± standard deviations (error bars) for three replicates.

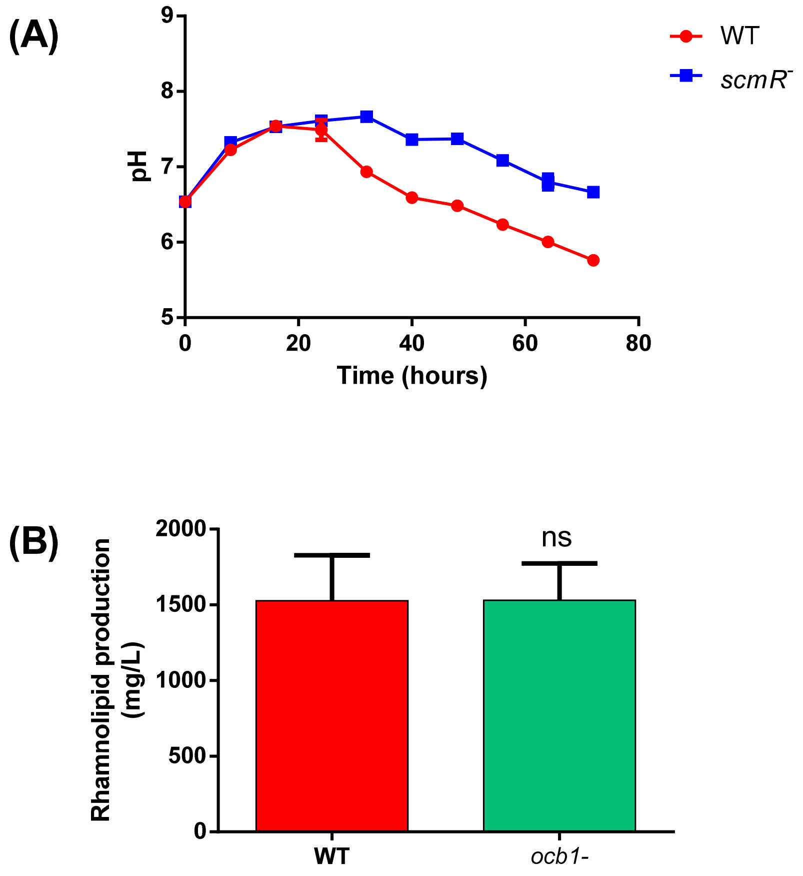

Supplementary Figure S4: Effect of pH on rhamnolipid biosynthesis. (A) pH was monitored in cultures of the *scmR*- mutant and the WT strain in NB medium supplemented with 2% glycerol at 30°C. (B)Rhamnolipid concentrations were measured in cultures of the *ocb1*- mutant and the WT strain. The error bars represent standard deviation from the mean (n = 3 independent cultures). Data analysed using a one-way ANOVA with post hoc. Dunnett's multiple comparisons tests (****$p < 0.0001$, ***$p < 0.001$, **$p < 0.01$, *$p < 0.05$, ns = not significant).

Supplementary Table 1: The transposon is inserted in the BTH_I1403 locus for M63 mutant.

TnSeq sequence	Percentage identity	Locus
**TCAGCGCCTCGAGCTGATCGAATTCCTCGAGGAGCGCCCGGC<br>AGCCGTCGAGGTAGCGGATGCCCGCTTCCGTCAGCGACAGAT<br>TGCGTGTCGTGCGATGGATGAGACGCGTGTTCAGATGTGTTTC<br>GAGCATCGCGATCGAACGAGATCG**	100%	BTH_I1403
